# Quantitation of cell-free DNA and RNA in plasma during tumor progression in rats

**DOI:** 10.1186/1476-4598-12-8

**Published:** 2013-02-04

**Authors:** Dolores C García-Olmo, María G Picazo, Inmaculada Toboso, Ana I Asensio, Damián García-Olmo

**Affiliations:** 1 Experimental Research Unit, General University Hospital of Albacete, C/ Hermanos Falcó 37, 02006, Albacete, Spain; 2 Department of Surgery, Universidad Autónoma de Madrid and La Paz University Hospital, IdiPaz, Madrid, Spain

**Keywords:** Cell-free nucleic acids, Plasma, Quantitation, Tumor size, Metastasis

## Abstract

**Background:**

To clarify the implications of cell-free nucleic acids (cfNA) in the plasma in neoplastic disease, it is necessary to determine the kinetics of their release into the circulation.

**Methods:**

To quantify non-tumor and tumor DNA and RNA in the plasma of tumor-bearing rats and to correlate such levels with tumor progression, we injected DHD/K12-PROb colon cancer cells subcutaneously into syngenic BD-IX rats. Rats were sacrificed and their plasma was analyzed from the first to the eleventh week after inoculation.

**Results:**

The release of large amounts of non-tumor DNA into plasma was related to tumor development from its early stages. Tumor-specific DNA was detected in 33% of tumor-bearing rats, starting from the first week after inoculation and at an increasing frequency thereafter. Animals that were positive for tumor DNA in the plasma had larger tumors than those that were negative (p = 0.0006). However, the appearance of both mutated and non-mutated DNA fluctuated with time and levels of both were scattered among individuals in each group. The release of non-tumor mRNA was unaffected by tumor progression and we did not detect mutated RNA sequences in any animals.

**Conclusions:**

The release of normal and tumor cfDNA into plasma appeared to be related to individual-specific factors. The contribution of tumor DNA to the elevated levels of plasma DNA was intermittent. The release of RNA into plasma during cancer progression appeared to be an even more selective and elusive phenomenon than that of DNA.

## Introduction

The presence of large quantities of cell-free nucleic acids (cfNA) in the serum and plasma of cancer patients was first reported several decades ago and was later verified for a variety of tumors (for review, see ref. [1]). In addition, it has been repeatedly demonstrated that some cfNA originate in tumors [2-4].

The presence of cfNA in plasma has been analyzed for its potential prognostic value in the diagnosis and management of cancer (for reviews, see refs. [1,5]). Analyses have been based on both qualitative and quantitative investigations of cfNA and, in particular, of cell-free DNA (cfDNA). However, the diversity of patients, tumors and laboratory techniques involved in such studies has resulted in divergent results. Thus, in spite of many apparently encouraging reports, the analysis of cfNA in plasma has not yet been translated into clinical practice.

Levels of cfDNA in plasma have been examined in cancer patients [4,6-10] and in tumor-bearing animals [4,8,11-15] but the results have been heterogeneous with respect to correlations of the levels of such DNA with tumor stage, location, size and/or response to therapy. Nonetheless, Diehl *et al.* proposed that quantitation of circulating mutant DNA, as a method for noninvasive monitoring of the course of therapy, might be useful in patients with metastatic colorectal cancer [4].

Quantitation of total cfDNA (both mutated and non-mutated) in plasma is easier than quantitation of tumor DNA in plasma, and the former has been proposed as a surveillance tool for cancer [9,16] and, even, for confirmation of the presence of primary tumors [9]. However, levels of total cfDNA in plasma do not appear to be useful for diagnostic screening [17,18], perhaps because overlapping concentrations of cfDNA are found in healthy individuals and in individuals affected by other pathological processes, for example, inflammation and trauma [1]. In spite of such limitations, some authors have suggested that accurate quantification of total DNA in the plasma of lung cancer patients, once a cutoff value has been established, might be a valuable tool for prognostic stratification [19] and for discrimination between patients with disease and unaffected individuals, and might be a useful strategy for early detection [20].

Non-tumor cfDNA has also been examined in both human and animal models [6-8,11,19]. In a previous study, we found that large amounts of non-tumor DNA were released during all stages of tumor progression in rats and, in particular, at early stages [11]. Our findings support the hypothesis that active interactions occur between tumor cells and non-tumor cells and are associated with the release of cfDNA during tumorigenesis.

Subsequent to the discovery of cfDNA in plasma, cell-free RNA (cfRNA) was also found in the plasma of cancer patients [21,22]. In theory, cfRNA should be much more fragile than cfDNA and should be more susceptible to degradation. Moreover, it has been reported that levels of ribonuclease are elevated in the serum of patients with pancreatic cancer [23]. However, cfRNA has been detected in plasma from patients with malignant melanoma [22], breast cancer [24], colon cancer [25,26], and gastric cancer [27], among others. Indeed, cfRNA has been proposed as a promising marker of cancer. By contrast, the authors of a recent study have suggested that detection of tumor-specific cfRNA is not a reliable tool for monitoring disease status in patients with neuroblastoma [28]. Nonetheless, in spite of the presence of degradative enzymes, not only is cfRNA present in plasma but it is also sufficiently intact to allow analysis by reverse transcription-PCR (RT-PCR; for review, see ref. [1]).

The kinetics of the release of cfNA into the bloodstream remain poorly understood and new approaches are needed since such kinetics might be critical to the interpretation of the variety of results obtained in previous studies in humans. The use of animal models for the relevant kinetic analyses is advantageous because of the uniformity of animals and tumors that might be expected to eliminate confounding factors.

The goal of the present study was to quantify, simultaneously, both tumor and non-tumor DNA and RNA in the plasma of tumor-bearing rats during tumor progression, from one week after the inoculation of tumor cells to the development, eleven weeks later, of metastases.

## Materials and methods

### Cell culture

We used DHD/K12-PROb cells (rat colon cancer cells; also called DHD/K12-TRb cells; European Collection of Cell Cultures no. 90062901; referred to as DHD cells herein) as malignant cells. DHD cells harbor a point mutation (G12D) in the *k-ras* oncogene [29]. Detection of this mutation during our analyses was considered equivalent to the detection of tumor DNA.

Cells were cultured in monolayer in a mixture of DMEM and Ham’s F10 (1:1, v/v; Gibco-BRL, Life Technologies Ltd., Paisley, Scotland), supplemented with 10% fetal bovine serum (Gibco-BRL) and gentamycin (0.005%; Gibco-BRL). Cells were passaged after dispersion in 0.125% trypsin in EDTA.

### Animals

We used both male and female BD-IX rats. They were taken from a colony established at the authors’ animal facility from founders purchased from a commercial breeder (CRIFFA, Barcelona, Spain). Breeding was performed in compliance with European Community Directive 86/609/CEE and Spanish law (Real Decreto 1201/2005) for the use of laboratory animals, which were the laws in force at the time of the animal experiments. The animal cancer model and the procedure for blood sampling were approved by the Animal Experimentation Ethics Committee of the University of Castilla-La Mancha, Spain (approval no. 0804.02). Personnel who took care of animals had been appropriately trained and accredited by the regional government.

As recommended by the Federation of European Laboratory Animal Science Associations (FELASA), rats in the animal facility were tested periodically to ensure that the colony remained free of pathogens.

From birth to the end of the experiments, all rats had unlimited access to water and standard rat chow (2014 Teklad Global; Harlan Laboratories, Indianapolis, IN, USA). At the beginning of the experiments, rats were six to eight weeks old.

### Implantation of tumors and design of experiments

Tumors were generated in the thoracic region by unilateral subcutaneous injection of DHD cells into the right side of the chest. Cells were trypsinized, washed, and resuspended in phosphate-buffered saline (PBS). Then 0.25 ml of this suspension, containing 1 x 10^6^ cells, was injected per rat.

After injection, each rat was randomly assigned to one of six groups. Groups were designated according to the time between the subcutaneous injection of cells and euthanasia, as follows (numbering of groups corresponds to numbers of weeks after injection): group 1, sacrificed one week after injection (n = 16); group 3, sacrificed three weeks after injection (n = 13); group 5, sacrificed five weeks after injection (n = 15); group 7, sacrificed seven weeks after injection (n = 16); group 9, sacrificed nine weeks after injection (n = 16); and group 11, sacrificed eleven weeks after injection (n = 14). Each group was composed of male and female animals in similar proportions.

Animals were inspected daily by the veterinarian of the animal facility. The body weight and the growth of subcutaneous tumors were monitored and recorded weekly. The greatest diameter of each tumor was measured with electronic calipers. Our previous experience with this model [11,29-31] demonstrated that higher-frequency analysis is unnecessary. The protocol for handling of tumor-bearing animals was designed according to recommendations for ethical cancer research [32].

In addition, we included a group of healthy BD-IX rats (n = 7), from which blood samples were taken and processed in the same way as those from tumor-bearing animals.

### Collection of blood and tissue samples

On the schedule indicated above, rats were anesthetized with an intraperitoneal injection of a mixture of ketamine (75 mg/kg) and xylazin (10 mg/kg). Then blood (approximately 4.0 to 4.5 ml per rat) was withdrawn by cardiac puncture and a lethal dose of sodium thiopental was administered intracardially immediately afterwards. All samples of blood were collected in tubes with EDTA and subjected to centrifugation (1,800 x g, 10 min, 4°C). Plasma was collected and subjected to a second centrifugation at 3,000 x g for 10 min. The plasma samples were divided into aliquots and stored at -80°C prior to extraction of DNA and RNA.

Lungs were inspected visually for the presence or absence of pulmonary metastases, which was recorded.

### Extraction of cfDNA and cfRNA from plasma samples

DNA was extracted from 240-μL samples of plasma with a commercial kit (NucleoSpin® Plasma XS; Macherey-Nagel, Düren, Germany), according to the instructions from the manufacturer. DNA samples were stored at -20°C.

RNA was extracted from 1-ml samples of plasma by a three-step procedure. First, cell-free nucleic acids (cfNA) were extracted with a commercial kit (QIAmp UltraSens Virus; Qiagen, Hilden, Germany). Then samples were subjected to incubation with DNase I (Qiagen) for 10 to 15 min. Finally, total RNA was treated with the RNeasy® MinElute® Cleanup kit (Qiagen) and samples of RNA were stored in RNase-free tubes at -20°C.

### Analysis of the purity of cfRNA and reverse transcription

To ensure the absence of DNA from the samples of RNA, we performed PCR to amplify a 165-bp sequence of the rat *k-ras* gene. The forward primer for PCR (sequence: 5'-ccctttacaaattgtacataga-3’) hybridized to an intronic sequence such that any observed amplification would be due to the presence of *k-ras* DNA.

Reverse transcription was performed with a commercial kit (Transcriptor High Fidelity cDNA Synthesis; Roche Diagnostics GmbH, Mannheim, Germany), using a protocol based on the use of random hexameric primers and the instructions from the manufacturer.

### Amplification and quantitation of cell-free nucleic acids in plasma samples by real-time PCR

DNA samples [both genomic DNA (gDNA) and cDNA] were analyzed by two real-time PCRs with fluorescent hybridization probes to amplify, respectively, total (mutated and non-mutated) *k-ras* sequences and exclusively mutated *k-ras* sequences. In the latter case, we used a peptide nucleic acid (PNA) probe, as a PCR-clamp, to suppress the amplification of the wild-type *k-ras* sequence so that only mutated *k-ras* sequences would be amplified. When no mutated *k-ras* was detected, we assumed that amplification of total DNA corresponded to the amplification of non-mutated DNA only. Samples in which mutated DNA was detected were excluded from the analysis of levels of non-mutated DNA.

**Figure 1:**
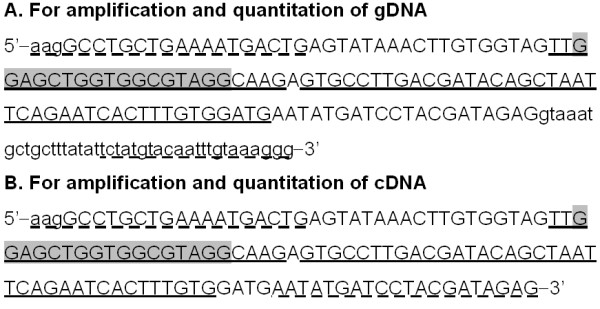
**Sequences of the *k-ras *gene that were amplified in the present study. A**) Sequence of genomic DNA (gDNA). **B**) Sequence of cDNA. Capital letters, Exon sequences; discontinuously underlined letters, sequences of the primers; underlined letters, sequences of fluorescent hybridization probes; shaded letters, sequence of the PNA probe.

For both sets of real-time PCRs, we used the same set of primers to amplify a specific fragment of exon 1 of the *k-ras* gene. For gDNA, we amplified a 165-bp sequence (Figure 1-A) with the primers forward, 5´-aaggcctgctgaaaatgactg-3’; and reverse, 5’-ccctttacaaattgtacataga-3’ (discontinuously underlined letters in Figure 1-A). For cDNA, we amplified a 125-bp fragment (Figure 1-B) with the same forward primer and the reverse primer 5’-ctctatcgtaggatcatattcatc-3’ (complementary to the last stretch of discontinuously underlined letters in Figure 1-B).

We used two hybridization probes for detection and quantitation of both total and mutated gDNA. One of these probes, the sensor probe, was designed to be complementary to the point mutation (GGT → GAT) in the *k-ras* oncogene (codon 12) that is found in DHD cells. The sensor probe was labeled at the 5’ end with LC-Red705 dye and phosphorylated at the 3’ end to yield the following sequence: 5’-LC-Red705-ttgcctacgccatcagctccaa-P-3’ (first stretch of underlined letters in Figure 1). We proved previously that this probe also hybridizes with non-mutated DNA templates. The sequence of the anchor probe, which was labeled at the 3’ end with fluorescein (F1), was 5´-catccacaaagtgattctgaattagctgtatcgtcaaggcact-F1-3’ (complementary to the last stretch of underlined letters in Figure 1-A). For the detection and quantitation of cDNA we used the same sensor probe and the anchor probe 5’-cacaaagtgattctgaattagctgtatcgtcaaggcac-F1-3’ (complementary to the last stretch of underlined letters in Figure 1-B). This probe hybridizes only with exonic sequences. Probes were manufactured by TIB MOBIOL (Berlin, Germany).

For the second amplification by PCR, designed to detect mutant *k-ras* exclusively, we added a PNA oligomer that encompassed codons 10 to 14 of exon 1 of the *k-ras* gene and had the sequence 5´-NH-cctacgccaccagctcc-CONH-3’ (complementary to shaded letters in Figure 1). This probe was obtained from Panagene (Daejeon, Korea).

For both sets of PCRs, we used an LC Fast Start DNA Master^PLUS^ Hyb Probes kit that included FastStart Taq DNA polymerase, reaction buffer, magnesium chloride and deoxynucleotide triphosphate mixture (Roche Diagnostics GmbH). The reaction mixture, with a total volume of 20 μl, contained a minimum of 2 ng of template gDNA in a volume of 2 μl or a minimum of 5 ng of template cDNA in a volume of 5 μl. It also contained 0.5 μM each primer, 0.15 μM sensor probe, 0.3 μM anchor probe and 5 mM MgCl_2_. For detection of mutated *k-ras*, PNA was added at a final concentration of 1 μM. For detection of mutated cDNA the procedure was similar to that described for the detection of non-mutated cDNA sequences with the exception that the volume of cDNA in the reaction mixture was 3 μl.

In all amplification experiments, DNA from DHD cells and water were included as positive and negative controls, respectively. gDNA or cDNA from the plasma of healthy animals was also included as a positive control in amplifications without the PNA probe and as a negative control in the case of PCR clamping.

We performed all PCRs with the LightCycler® System (Roche Diagnostics), using LightCycler® Software (version 3.5). Samples were placed in capillary tubes and subjected to initial denaturation by incubation at 95°C for 10 sec. Then amplification was allowed to proceed for 50 cycles of denaturation, annealing and extension. The first step in the cycle was incubation for 2 sec at 95°C. The annealing incubation was allowed to proceed for 7 sec at 53°C in the case of gDNA and for 10 sec at 58°C in the case of cDNA. Finally, extension was allowed to proceed for 15 sec at 72°C. During the second incubation (annealing step), fluorescence was monitored at 705 nm (F3 channel in the LightCycler® system).

For detection of mutated *k-ras*, we included an additional incubation (70°C, 10 sec) between the denaturation and the annealing steps, for the binding of PNA.

Each reaction was performed in triplicate. We generated four standard curves for the quantitation of, respectively, non-mutated and mutated 165-bp *k-ras* sequences, and non-mutated and mutated 125-bp *k-ras* sequences. In this way we were able to quantify mutated and non-mutated DNA and RNA (cDNA). Each curve was created by plotting the logarithm of the amount of DNA against the threshold cycle number. The amounts of gDNA or cDNA in samples to be analyzed were determined from the appropriate standard curves.

The amounts obtained by the above-described method were recalculated to generate concentrations (number of DNA fragments/ml of plasma) using the following formulas:

– For quantitation of plasma DNA: [(Quantity of gDNA obtained by PCR in fg/ Molecular mass of the sequence in fg) / Volume of gDNA template in the PCR reaction in μl] x (Total volume of gDNA at the end of the extraction from plasma in μl / Volume of plasma used for DNA extraction in ml).

– For quantitation of plasma RNA: [(Quantity of cDNA obtained by PCR in fg/ Molecular mass of the sequence in fg) / Volume of cDNA template in the PCR reaction in μl] × (Total volume of cDNA at the end of the RT reaction in μl / Volume of plasma used for RNA extraction in ml).

For detection of mutated gDNA or cDNA, samples were subjected to two identical consecutive amplifications by PCR with the PNA probe. A plasma sample was considered positive when at least one replicate of the two PCRs yielded the mutated *k-ras* sequence.

### Assessment of the sensitivity of the PCR-based techniques for detection of mutated k-ras sequences

We evaluated the sensitivity of the method for detection of the *k-ras* mutation by diluting DNA from DHD cells, which carry the G12D mutation, with DNA extracted from the plasma of healthy animals. We made serial dilutions, from 1:10 to 1:1000. The 1:10 mixture contained 1 ng of mutant DNA and 10 ng of wild-type DNA. Similarly, we made serial dilutions of RNA from DHD cells with RNA extracted from the plasma of healthy animals.

### Statistical analysis

Comparisons of means were made by Student’s t-test for normal distributions and by the Kruskal-Wallis tests for non-normal distributions. A p-value of less than 0.05 was accepted as evidence of statistical significance. Qualitative variables were compared using the χ^2^ test and Fisher’s exact test when any frequency was less than 5. Two-tailed p-values of less than 0.05 were considered evidence of statistical significance.

The relationships between the amounts of DNA and RNA in the plasma and (i) tumor size and (ii) the presence of metastasis were determined by Pearson correlation analysis. We also analyzed the relationship between quantities of DNA and RNA. Statistical analyses were performed with StatPlus® version 2009 (AnalystSoft) and OpenEpi (Open Source Epidemiologic Statistics for Public Health, Version 2.3.1, http://www.OpenEpi.com).

## Results

### Growth of tumors

**Figure 2:**
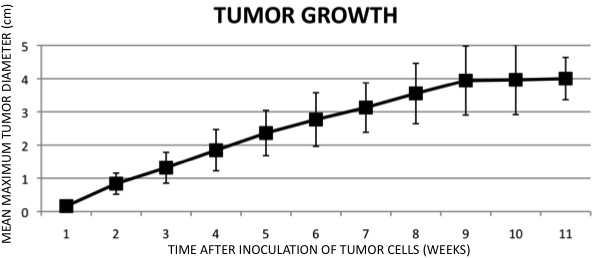
**Growth of tumors in rats after subcutaneous injection of DHD cells.** Values shown are mean maximum diameters of tumors (±S.D.) for all animals examined.

Tumors were detectable in rats from the first week after inoculation of DHD cells and grew at an apparently uniform rate for the duration of the experiment (Figure 2). There were no statistically significant differences between tumors in male and female rats.

### Macroscopic observations

No metastases were observed in the abdominal tissues of any of the rats; metastases were only found in the lungs and lymphatic tissues. Lung macrometastases were detected in five animals in group 7 (5/16; 31%), in eight animals in group 9 (8/16; 50%), and in nine animals in group 11 (9/14; 64%). In male rats, the presence of lung macrometastases was slightly more frequent than in females (p = 0.049).

### Analysis of plasma DNA by real-time PCR

First, we assessed the sensitivity of the method for detection of a mutation in the *k-ras* gene by dilution experiments with various amounts of mutant DHD DNA and wild-type DNA from rat plasma. The clamped probe assay detected mutated DNA even when there was a 100-fold excess of wild-type plasma DNA.

**Figure 3:**
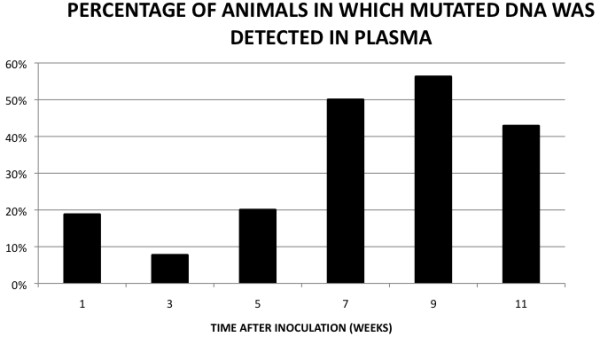
Histograms showing the percentage, in each group (as indicated by time in weeks), of animals that was positive for tumor (mutated) DNA in plasma.

DNA was extracted from plasma from all animals and analyzed. Tumor DNA (mutated *k-ras* sequences) was found in three of the plasma samples from animals in group 1 (3/16), in one in group 3 (1/13), in three in group 5 (3/15), in eight in group 7 (8/16), in nine in group 9 (9/16), and in six in group 11 (6/14; Figure 3). Thus, we detected tumor DNA in plasma of 30 out of the 90 tumor-bearing animals in the study (33%), and, in particular, we detected tumor DNA in 11 of the 22 animals that had lung macrometastases (50%). However, the relationship between the detection of mutated DNA in plasma and the presence of metastases was not statistically significant (p = 0.07).

The presence of *k-ras* mutated sequences in primary tumors and lung metastases from rats injected with DHD cells was confirmed in previous studies [31]. Nonetheless, we tested lung metastases from five rats whose plasma samples were negative for the detection of mutated *k-ras* sequences. After amplification by PCR with the PNA probe, we detected such mutated sequences in all five samples.

The mean tumor diameter in animals in which tumor DNA was detected in plasma was 3.4 ± 1.7 cm (n = 30), whereas in animals in which no tumor DNA was detected in plasma the mean diameter was 2.1 ± 1.6 cm (n = 60). This difference was statistically significant (p = 0.0006).

**Figure 4:**
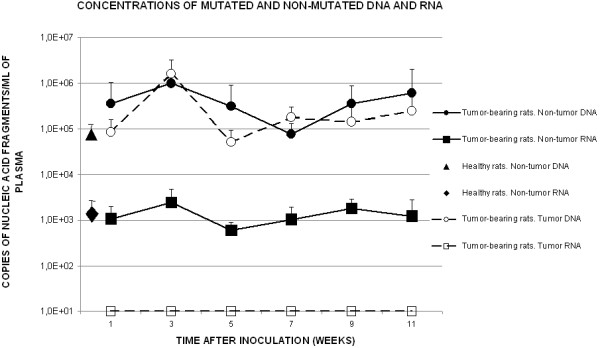
**Amounts of DNA and RNA, as detected by real-time PCR, in plasma from healthy and tumor-bearing rats at different stages of tumor progression.** Values are means of results from all rats in each group with the exception of rat no. 7-5, in the case of levels of tumor DNA, and of rat no. 9-10, in the case of levels of non-tumor RNA, as discussed in the text.

The concentration in plasma of the mutated *k-ras* sequence ranged from 1.8 × 10^2^ to 1.6 × 10^6^ fragments/ml of plasma (2.1 × 10^5^ ± 3.5 × 10^5^ fragments/ml; mean ± s.d.; n = 29). In one animal in group 7 (no. 7-5), the concentration was very high, namely, 1.9 × 10^8^ fragments/ml of plasma. Since this concentration exceeded the mean value by three orders of magnitude, it was excluded from the statistical analyses of levels of tumor DNA. Statistical comparisons between groups did not reveal any statistically significant differences. The means of values obtained from each group are shown in Figure 4.

In the samples of plasma in which no mutated DNA was detected, the first amplification of total DNA (without the PNA probe) was considered to correspond to both the amplification and the quantitation of non-mutated DNA.

The mean concentrations of non-mutated *k-ras* sequences in plasma from healthy animals ranged from 1.5 × 10^3^ to 1.8 x 10^5^ fragments/ml of plasma (9.0 x 10^4^ ± 7.7 × 10^4^ fragments/ml; n = 7). In samples from tumor-bearing rats, the concentrations ranged from 1.5 x 10^3^ to 5.8 × 10^6^ fragments/ml of plasma (4.7 × 10^5^ ± 1.2 × 10^6^ fragments/ml; n = 60). The means of values obtained from each group are shown in Figure 4.

Overall, levels of non-mutated DNA in all tumor-bearing animals together were higher than those in healthy animals, and the difference was statistically significant (p = 0.02). However, there was no statistically significant difference between the group of healthy animals and each group of tumor-bearing rats. The lowest mean value was found in group 7, although there was no statistically significant difference with respect to the values in other groups. The standard deviations in the concentrations of non-tumor DNA in tumor-bearing rats were higher than those of tumor DNA in these rats.

Pearson correlation analysis showed that, overall, the concentrations of both mutated and non-mutated *k-ras* sequences in plasma were unrelated to tumor size. However, concentrations of mutated *k-ras* sequences were slightly higher in animals whose tumors were equal to or greater than 2 cm in diameter than in animals with tumor diameters of less than 2 cm (p = 0.045).

Student’s t-test showed that there were no significant differences in the concentrations of both mutated and non-mutated *k-ras* sequences between animals with and without macrometastases.

### Quantitation of plasma RNA by real-time PCR

We were able to analyze RNA in only some of the animals since volumes of plasma were not always sufficient for all analyses. Specifically, we analyzed plasma RNA from eight animals each in groups 1, 3 and 9; from seven animals each in groups 5 and 11; from six animals in group 7 (i.e., from 44 tumor-bearing rats in total), and from five healthy animals.

In seven plasma samples (15%), no *k-ras* cDNA was detected. There was one such negative sample in each of groups 1, 3, 7 and 9 and in the group of healthy rats; there were two negative samples in group 5 and none in group 11.

We failed to find mutated RNA sequences in any of the plasma examined. Thus, the first amplification without the PNA probe was considered to correspond to both the amplification and the quantitation of non-mutated cDNA. We assessed the sensitivity of the method for detection of mutated sequences of *k-ras* RNA by dilution experiments with various amounts of mutant DHD RNA and wild-type plasma RNA. The assay detected mutated RNA sequences even when there was a 1000-fold excess of wild-type plasma RNA.

In the samples from healthy animals, the concentrations of non-mutated *k-ras* cDNA ranged from 610 to 3,100 fragments/ml of plasma (1,400 ± 1,200 fragments/ml; n = 4). In samples from tumor-bearing rats, the concentrations ranged from 23 to 6,100 fragments/ml of plasma (1,400 ± 1,400 fragments/ml; n = 36). The mean values obtained from each group are shown in Figure 4. The result for one animal in group 9 (no. 9-10) was excluded from the analysis since the concentration (150,000 fragments/ml) was two orders of magnitude higher than that for the animal that, otherwise, gave the highest result. There was no statistically significant difference between healthy and tumor-bearing animals.

Pearson correlation analysis showed that, overall, the concentration of non-mutated RNA was unrelated to tumor size, and Student’s t-test showed that there was no significant difference between animals with and without macrometastases.

The mean concentration of non-mutated RNA in plasma was lower than that of non-mutated DNA in plasma in both tumor-bearing (p = 0.004) and healthy rats (p = 0.04).

## Discussion

The clinical value and role of cfNA in plasma of cancer patients remain to be clarified (for review, see ref. [1]). However, release of cfNA from tumors might be directly involved in the development of metastases, as proposed in the “Genometastasis Hypothesis” [30,33], and it has been reported that plasma DNA can, indeed, transfect and oncogenically transform cultured cells [34].

The kinetics of changes in the concentration of cfNA in plasma during cancer progression have not been fully defined and previously reported results are non-uniform. Therefore, in the present study, we used a heterotopic rat model of colon cancer, in which tumors were generated subcutaneously, and real-time PCR to quantify non-mutated and mutated DNA and RNA sequences in plasma from both healthy and tumor-bearing animals as tumors developed. In a similar previous study, we focused on the release of non-tumor DNA, using a similar method [11]. In the present study, we increased the number of animals examined in order to focus on mutated DNA, and we used real-time RT-PCR to detect and quantify RNA fragments in plasma.

We detected non-mutated *k-ras* sequences in plasma from all healthy and cancerous animals, and we detected mutated *k-ras* sequences in 33% of all tumor-bearing rats examined. Concentrations both of tumor and non-tumor DNA in plasma fluctuated as tumors developed.

The results of quantitation of non-mutated *k-ras* sequences confirmed those in our previous study [11]. Overall, we found that concentrations of circulating non-mutated *k-ras* sequences were significantly higher in tumor-bearing animals than in healthy animals (p = 0.02). However, when we compared, individually, each of the six groups of tumor-bearing rats with healthy animals, there were no statistically significant differences as a consequence of the high standard deviations in the groups of tumor-bearing rats. Such fluctuations and scattering of values, which appear to be inherent to the release of DNA into the bloodstream, might be one of the reasons for the difficulties encountered in efforts to establish standard cut-off levels for diagnosis and prognosis in a clinical setting.

Most previous human studies have found similarly elevated levels of total DNA [9,16] and/or non-tumor DNA [6,7] in plasma from cancer patients, but relationships between such levels and tumor size and other clinical parameters remain contradictory [7,9,35-38]. It is possible that the kinetics of release of cfDNA might be specific, to a first approximation, to each kind of cancer. For example, two studies, using identical methods, showed that levels of cfDNA in plasma from patients with esophageal cancer were higher than those in patients with gastric cancer. Moreover, in the latter patients, levels were associated with tumor stage, which was not the case in the patients with esophageal cancer [6,7].

In the present study, diameters of tumors were not correlated with levels of non-tumor DNA in plasma. In fact, while tumors were growing at a steady rate (Figure 2), concentrations of non-mutated DNA in plasma fluctuated, with a peak at the early stage of tumor progression (three weeks after the inoculation of cells; Figure 4). The timing of this release of non-tumor cfDNA was very similar to that reported some years ago in the same animal model [11], and the present results also support the hypothesis that plasma levels of non-mutated DNA might be a particularly useful diagnostic tool at the early stages of tumor development. We do not know why the concentration of non-mutated DNA in plasma is significantly elevated at the early stages of tumor progression. At these early stages, normal and malignant cells interact and their interactions might lead to the release of non-tumor DNA via, perhaps, enhanced turnover of cells and cell death or even the active release of DNA from host cells, as demonstrated previously in cultured cells [39]. In a recent study, the authors supported the notion that the release of non-tumor cfDNA to the bloodstream is related to cancer progression, since their results showed a potential prognostic power of the quantitation of such DNA in plasma from lung cancer patients [19].

In the present study, detection of mutated DNA in plasma was an early event, occurring from the first week after inoculation of cells, and both the frequency of such detection and the levels of mutated DNA increased with time and tumor diameter. The animals in which tumor DNA was detected in plasma had larger tumors than those in which it was not (p = 0.0006), and animals with tumors of 2 cm or more in diameter had slightly higher levels of tumor DNA in their plasma than animals with smaller tumors (p = 0.045). However, there was no statistically significant correlation between variables. In addition, the frequency of tumor DNA-positive plasma in animals with metastases was only 50%, and levels of tumor DNA in these animals were not significantly different from those in animals without metastases. These results are in agreement with those of previous studies of patients with colorectal cancer, which failed to demonstrate any significant correlation between tumor load (including metastatic deposits) and the amount of mutant DNA in the circulation [40]. Our failure to detect the mutated *k-ras* sequence in half of our animals with metastases cannot be explained by the hypothetical absence of this mutated sequence in metastases. The presence of mutated *k-ras* sequences in both the primary tumors and the lung metastases from rats injected with DHD cells was confirmed in previous studies [31] and, in the present study, we examined a number of lung metastases and all yielded positive results with respect to the detection of such mutated sequences.

The highest frequency of detection of tumor DNA in plasma was 56% (at the ninth week after inoculation). This relatively low frequency supports the hypothesis that increases in concentrations of total DNA in plasma during cancer progression are not due to DNA from neoplastic cells, as also suggested by previous studies in patients with breast [35] and colorectal cancer [40]. To explain the absence or low levels of mutant DNA in plasma after cancer has progressed, it has been proposed that the fragments of tumor DNA are degraded more effectively in plasma than the circulating DNA derived from non-neoplastic cells [40]. By contrast, a recent study of colorectal cancer patients indicated that concentrations of total cfDNA and mutant *KRAS* DNA in plasma were strongly correlated, leading the authors to postulate that the increasing concentrations of cfDNA in the bloodstream of cancer patients are primarily of tumor origin [10]. The results of the present study suggest that the contribution of tumor DNA to the increased concentrations of plasma DNA in cancer patients might be intermittent, and, moreover, that DNA derived from normal and tumor cells might behave differently in terms, for example, of susceptibility to degradation.

In studies with animal models, some authors have found increasing concentrations of tumor DNA in plasma from xenografted immunodeficient mice [12-15], with a positive correlation between concentrations of tumor-derived DNA in plasma and the size of tumors [12,14,15]. A similar observation was reported in CD1 mice that had been injected with CD1-derived tumor cells and maintained for two weeks [8]. These results are not fully confirmed by the present study since the concentrations that we determined were more unstable, perhaps because of some major differences in experimental design with respect to number of animals and duration. In the present study, we used immunocompetent rats injected with rat tumor cells (allografted animals), and this experimental design results in interactions between tumor and host cells that are probably closer to those in human malignancy than are the interactions in immunodeficient animals.

With respect to the non-mutated mRNA in plasma, concentrations were similar in healthy and tumor-bearing rats, remaining basically unchanged during tumor progression (Figure 4). Concentrations of mRNA were also lower than those of non-tumor DNA in healthy rats (p = 0.04), as well as in tumor-bearing ones (p = 0.004).

It has been reported that concentrations of specific RNA sequences exclusively are selectively elevated in cancer patients. For example, levels of β-catenin mRNA were found to be selectively elevated in plasma of patients with colorectal carcinomas and adenomas, but levels of glyceraldehyde-3-phosphate dehydrogenase (GAPDH) mRNA were not [41]. Note, in this context, that immunostaining of β-catenin usually reveals abnormal nuclear accumulation of this protein in tumor cells. Similarly, it has been reported that thymidylate synthase (TS) mRNA was detected more frequently and at higher levels in plasma of patients with colorectal cancer than in plasma of healthy subjects [42]. In addition, a statistically significant correlation was found between high levels of TS in tumors and the presence of TS mRNA in plasma [42]. Moreover, the activity of human telomere-specific reverse transcriptase (hTERT) is elevated in most colorectal tumors and the presence in plasma of hTERT mRNA has been proposed as a marker for detection and monitoring of colorectal cancer [26,43]. In the present study, mutated *k-ras* mRNA sequences (derived from tumor cells) were not detected in any animal examined. This observation suggests that the release of tumor-derived RNA into plasma is a more elusive phenomenon than the release of DNA and is probably restricted to overexpressed sequences in each kind of tumor.

It has been proposed that extracellular plasma RNA is confined to vesicle-like structures, for example, exosomes [44,45]. Balaj *et al*. proposed recently that tumor microvesicles might contain not only elevated levels of RNA, but also DNA, mutated and amplified oncogene sequences and transposable elements [46]. Exosomes have been described as particles that are able to transfer their RNA content to cells, and it has been proposed that this RNA can be functional in its new location [47]. Such RNA might be related to the pro-metastatic capacity of exosomes. However, the possibility of horizontal transfer of DNA from such particles should not be ignored since there is strong evidence that DNA, circulating in plasma, might be transferred to susceptible cells, with the subsequent oncogenic transformation of these cells [34].

## Conclusions

In the present study of a homogeneous population of syngenic animals with a predictable malignancy, the release of cfDNA into plasma was greater and more frequent than that of cfRNA. The presence of detectable tumor DNA and of high levels of non-tumor DNA in plasma was related to the induced cancer from its early stages, whereas the release of non-tumor RNA was unaffected by tumor progression and the release of mutated RNA was undetectable. Concentrations of cfDNA in plasma fluctuated during tumor progression and were scattered among individuals, suggesting that the release of cfDNA was related to individual factors and even to the kind of DNA (normal or tumor DNA). The release of non-tumor DNA followed a specific time course that was predictable from the results of previous studies in the same model, but it would probably be very difficult to define such kinetics in humans with a view to clinical relevance because of the heterogeneity of individual patients and tumors, even in cases of the same kind of cancer.

Finally, our results suggest that the release of RNA and the release of DNA into the plasma are basically independent phenomena. Further studies are needed to examine whether, in spite of this independence, the two phenomena act cooperatively in tumor progression.

## Competing interests

The authors have no competing interests to disclose.

## Authors’ contributions

DCGO conceived the study and its design, coordinated the experiments, performed the analysis of results and drafted the manuscript. MGPM, IT and AIA carried out the experiments in laboratory animals and the assays by PCR. DGO participated in the design of the study and helped to draft the manuscript. All authors read and approved the final manuscript.
